# Gender Trouble in Social Psychology: How Can Butler’s Work Inform Experimental Social Psychologists’ Conceptualization of Gender?

**DOI:** 10.3389/fpsyg.2018.01320

**Published:** 2018-07-27

**Authors:** Thekla Morgenroth, Michelle K. Ryan

**Affiliations:** ^1^Department of Psychology, University of Exeter, Exeter, United Kingdom; ^2^Faculty of Economics and Business, University of Groningen, Groningen, Netherlands

**Keywords:** gender trouble, gender, gender performativity, social psychology, non-binary gender, genderqueer, Judith Butler

## Abstract

A quarter of a century ago, philosopher Judith Butler (1990) called upon society to create “gender trouble” by disrupting the binary view of sex, gender, and sexuality. She argued that gender, rather than being an essential quality following from biological sex, or an inherent identity, is an *act* which grows out of, reinforces, and is reinforced by, societal norms and creates the illusion of binary sex. Despite the fact that Butler’s philosophical approach to understanding gender has many resonances with a large body of gender research being conducted by social psychologists, little theorizing and research within experimental social psychology has drawn directly on Butler’s ideas. In this paper, we will discuss how Butler’s ideas can add to experimental social psychologists’ understanding of gender. We describe the Butler’s ideas from *Gender Trouble* and discuss the ways in which they fit with current conceptualizations of gender in experimental social psychology. We then propose a series of new research questions that arise from this integration of Butler’s work and the social psychological literature. Finally, we suggest a number of concrete ways in which experimental social psychologists can incorporate notions of gender performativity and gender trouble into the ways in which they research gender.

“We’re born naked, and the rest is drag.”

(RuPaul, 1996)

## Introduction

A quarter of a century ago, philosopher Judith Butler (1990) called upon society to create “gender trouble” by disrupting the binary view of sex, gender, and sexuality. Key to her argument is that gender is not an essential, biologically determined quality or an inherent identity, but is repeatedly *performed*, based on, and reinforced by, societal norms. This repeated performance of gender is also *performative*, that is, it creates the idea of gender itself, as well as the illusion of two natural, essential sexes. In other words, rather than *being* women or men, individuals *act* as women and men, thereby creating the categories of women and men. Moreover, they face clear negative consequences if they fail to do their gender right.

We argue that Butler’s philosophical approach to under standing gender has many resonances with, and implications for, a large body of gender research being conducted by social psychologists. Indeed, Butler’s notion of performativity echoes a range of social psychological approaches to gender and gender difference. What we social psychologists might call gender norms and stereotypes (e.g., Eagly, 1987; Fiske and Stevens, 1993), or gender schemas (Bem, 1981) provide the “scripts” for what Butler’s describes as the performance of gender.

We are not the first to point out the relevance of Butler’s work to social psychology. Bem (1995) drawing on Butler’s work, argued in that as gender researchers we should create *gender trouble* by making genders that fall outside of the binary visible, in order to disrupt binary, heteronormative views of gender within and outside of psychology. Minton (1997) argued that queer theory more broadly, which challenges the binary, heteronormative system of sex and gender, should inform psychological theory and practice. Similarly, Hegarty (1997) uses Butler’s arguments regarding performativity to criticize neuropsychological research that essentializes sexual orientation, pointing out the ways in which it ignores historical and cultural variation in sexuality and excludes women and other minorities. However, despite these calls for gender trouble over 20 years ago, we believe that social psychology, and experimental social psychology in particular, has yet to truly step up and answer the call.

Despite past acknowledgments of the importance of Butler’s work by social psychologists, in particular by qualitative psychologist, to our knowledge, little theorizing and research within experimental (and quantitative) social psychology has directly drawn on Butler’s ideas. This is despite the fact that there are identifiable similarities in broad theoretical ideas espoused by many social psychologists with an interest in gender and Butler’s ideas. Thus, we argue that there is great value in (again) promoting the ideas Butler puts forward in *Gender Trouble* to social psychologists. While experimental social psychological perspectives on gender have been concerned primarily with the origin and perpetuation of gender stereotypes, Butler’s work is more political in her explicit call to create gender trouble. The political nature of the work is perhaps one reason why experimental social psychologists have been reluctant to build on and integrate Butler’s ideas in their work – but, we would argue, it is indeed one of the reasons they should. Combining these two perspectives seems potentially fruitful, bringing together Butler’s theorizing and her call for social and political change with established experimental social psychological theory and empirically testable hypotheses.

In this paper we will first describe Butler’s work in more detail. We will then discuss the extent to which her work fits with different conceptualizations of gender in the social psychological literature, with a focus on experimental social psychology. We will then propose new avenues of research that could potentially grow out of an integration of Butler’s work into social psychology. Finally, we will discuss the different ways in which Butler’s work can inform and challenge the ways in which we, as experimental social psychologists, study and operationalize gender.

## Butler’s View on Gender

In her book *Gender Trouble* Butler (1990) argues that within Western culture, sex, gender, and sexual orientation are viewed as closely linked, essential qualities. The prevalent view is that biological sex is binary (male vs. female), essential, and natural, and that it forms the basis for binary gender, which is viewed as the cultural interpretation of sex, and sexual desire. In other words, there is a belief that a baby born with a penis will grow up to identify and act as a man – whatever that means in a specific culture – and, as part of this gender role, be sexually attracted to women. Similarly, there is a belief that a baby born with a vagina will grow up to identify and act as a woman and, as part of this gender role, be sexually attracted to men. Butler argues that these configurations of sex, gender, and sexual desire are the only “intelligible” genders in our culture.

This societal view of gender is also reflected in the works of many feminist writers, who define sex as biological and gender as cultural (see Gould, 1977, for a review and critical discussion). Butler criticizes this distinction between sex – as natural, essential, and pre-discursive (i.e., existing *before* culture and before interpretation) – and gender as its cultural interpretation. She argues that it is not just gender that is culturally constructed and has prescriptive and proscriptive qualities, but that this also applies to sex as a binary category. Through this, Butler (1990) argues that the distinction between sex and gender is meaningless, noting that “perhaps this construct called ‘sex’ is as culturally constructed as gender; indeed, perhaps it was always already gender with the consequence that the distinction between sex and gender turns out to be no distinction at all” (p. 9).

Butler cites evidence for the considerable variability in chromosomes, genitalia, and hormones, that don’t always align in the expected, binary manner. Indeed, even biologists, who traditionally view the body as natural and pre-discursive, increasingly argue that a binary view of human sex is overly simplistic and that sex should be viewed as a spectrum rather than a dichotomy, in terms of anatomical, hormonal, and even cellular sex (see Fausto-Sterling, 2000; Ainsworth, 2015 see also Fausto-Sterling, 1993). This variability can include ambiguous genitalia, a “mismatch” between chromosomes and genitalia, or a body that is comprised of a mix of “male” (XY) and “female” (XX) cells^1^. Some research suggest that up to 10% of children are born with sex characteristics that do not clearly fall into the category of female or male (e.g., Arboleda et al., 2014), although these numbers are debated and some argue the number is much lower. For example, Sax (2002) argues that only very specific “conditions” should qualify as intersex and that only about 0.018% of people should be considered intersex. We would argue, however, that exact numbers or specific definitions of what constitutes “intersex” are irrelevant here and that debates about exact numbers are indeed illustrative of the very process Butler discusses – that there is no “objective” or natural sex, but that it is performatively constructed.

Regardless of exact numbers, Butler argues that any individual who does not fall clearly into one of the two sex categories is labeled as abnormal and pathological (see Sax’s usage of the term “condition”), and steps are taken to “rectify” this abnormality. For example, the majority of babies born with intersex characteristics undergo surgery and are raised as either male or female (Human Rights Watch, 2017), protecting and maintaining the binary construction of sex.

To be clear, Butler does not argue that biological processes do not exist or do not affect differences in hormones or anatomy. Rather, she argues that bodies do not exist outside of cultural interpretation and that this interpretation results in over-simplified, binary views of sex. In other words, biological processes do not themselves result in two “natural,” distinct, and meaningful, categories of people. The two sexes only appear natural, obvious, and important to us because of the gendered world in which we live. More specifically, the repeated performance of two polar, opposite genders makes the existence of two natural, inherent, pre-discursive sexes seem plausible. In other words, Butler views gender as a performance in which we repeatedly engage and which creates the illusion of binary sex. She argues:

“Because there is neither an ‘essence’ that gender expresses or externalizes nor an objective ideal to which gender aspires; because gender is not a fact, the various acts of gender create the idea of gender, and without those acts, there would be no gender at all. Gender is, thus, a construction that regularly conceals its genesis. The tacit collective agreement to perform, produce, and sustain discrete and polar genders as cultural fictions is obscured by the credibility of its own production. The authors of gender become entranced by their own fictions whereby the construction compels one’s belief in its necessity and naturalness.” (p. 522)

Thus, for Butler, gender is neither essential nor biologically determined, but rather it is created by its own performance and hence it is *performative*. The term *performativity*, originating in Austin’s (1962) work on performative utterances, refers to speech acts or behaviors which create the very thing they describe. For example, the sentence “I now pronounce you man and wife” not only describes what the person is doing (i.e., pronouncing something) but also creates the marriage (i.e., the thing it is pronouncing) through the pronouncement. Butler builds on this work by exploring how gender works in a similar way – gender is created by its own performance.

However, as this binary performance of gender is almost ubiquitous, its performative nature is concealed. The binary performance of gender is further reinforced by the reactions of others to those who fail to adhere to gender norms. Butler argues that “Discrete genders are part of what ‘humanizes’ individuals within contemporary culture; indeed, those who fail to do their gender right are regularly punished” (p. 522). This punishment includes the oppression of women and the stigmatization and marginalization of those who violate the gender binary, either by disrupting the presumed link between sex and gender (e.g., transgender individuals) or between sex and sexuality (e.g., lesbian and gay individuals) or by challenging the binary system in itself (e.g., intersex, bisexual, or genderqueer individuals). This stigma is clearly evidenced by the high rate of violence against transgender women, particularly those of color (Adams, 2017); surgeries performed on intersex babies to achieve “normal” sex characteristics (Human Rights Watch, 2017); and the stigmatization of sexual minorities (Lick et al., 2013).

These negative reactions and the binary performance of gender, Butler argues, do not exist by chance. Instead, they serve as tools of a system of power structures which is trying to reproduce and sustain itself – namely a patriarchal system of compulsory heterosexuality in which women serve as a means of reproduction to men, as their mothers and wives. These power structures are both *prohibitive* (i.e., proscriptive), repressing deviating gender performance, as well as *generative* (i.e., prescriptive), creating binary, heteronormative gender performance.

Butler’s work is a call to action to overthrow these structures and end the problematic practices that they engender. However, she criticizes feminist voices who emphasize a shared identity (“women”) to motivate collective action on behalf of the group in order to achieve societal changes. By arguing that gender is not something one *is*, but rather something one *does* or *performs*, Butler argues that gender identity is not based on some inner truth, but instead a by-product of repeated gender performance. Framing gender identity as an inherent part of the self, as many feminist writers did at the time (and indeed still do), she argues, reinforces the gender binary and in turn plays into the hands of the patriarchy and compulsory heterosexuality. Feminists should instead seek to understand how the category of “women” is produced and restrained by the means through which social change is sought (such as language or the political system).

This argument has particular relevance to the notion of gender identity. As such, it has been criticized as invalidating transgender individuals, whose experience of a true inner gender identity that is not in line with the sex they were assigned at birth is often questioned. This is despite the fact that from a young age transgender individuals view themselves in terms of their expressed gender, both explicitly and implicitly, mirroring self-views of *cis*-gender^2^ children (Olson et al., 2015). Butler has responded to these criticisms repeatedly. For example, answering a question about what is most often misunderstood about her theory in an interview in 2015, she replies:

“I do know that some people believe that I see gender as a “choice” rather than as an essential and firmly fixed sense of self. My view is actually not that. No matter whether one feels one’s gendered and sexed reality to be firmly fixed or less so, every person should have the right to determine the legal and linguistic terms of their embodied lives. So whether one wants to be free to live out a “hard-wired” sense of sex or a more fluid sense of gender, is less important than the right to be free to live it out, without discrimination, harassment, injury, pathologization or criminalization – and with full institutional and community support.” (The Conversation Project, 2015)

Thus, Butler does not question people’s sense of self, but instead criticizes a shared gender identity as the necessary basis for political action. She points out that abandoning the idea of gender as an identity does not take away the potential of agency on behalf of women. Instead, it opens up the possibility of agency, which other approaches that view identity as fixed and stable do not enable. The fact that identity is *constructed* means that it is neither completely arbitrary and free, nor completely determined, leaving room for re-structuring, subversion, and for disrupting the status quo. Thus, the common identity “we, women” is not necessary for collective action on behalf of the feminist movement, as anyone can engage in subversion and the disruption of the gender binary. Indeed, we would argue that feminism becomes more powerful as an inclusive movement for gender equality more broadly defined, not just equality between women and men.

In conclusion, Butler argues that we, as a society, need to create gender trouble by disrupting the gender binary to dismantle the oppressive system of patriarchy and compulsory heterosexuality. While some of Butler’s ideas seem very different from how gender is generally viewed in the experimental social psychological literature, others resonate well with social psychological theorizing and empirical research. In the next section, we will discuss ways in which Butler’s view is compatible – and incompatible – with some of the most prominent conceptualizations of gender in experimental social psychology.

## Is Butler’s View Compatible With Conceptualizations of Gender in Social Psychology?

Gender has been an increasingly important focus within psychology more generally, and in social psychology in particular (e.g., Eagly et al., 2012). While there is considerable variation in how psychologists view and treat gender, we argue that many of approaches fall into one of three traditions: (1) evolutionary approaches which view binary, biological sex as the determinant of gender and gender differences; (2) social structural approaches which view societal forces such as status and social roles as the determinant of gender stereotypes and, in turn, gender differences; and, not mutually exclusive from a social structural approach; (3) social identity approaches which view gender as one out of many social categories with which individuals identify to varying degrees. In addition, integrative approaches draw on more than one of these traditions, as well as developmental, social cognitive, and sociological models of gender, and integrate them to explain gendered behavior. While none of these approaches is entirely compatible with the argument that binary sex is constructed through the repeated binary performance of gender with gender identity as a by-product of this performance, there are great differences in the extent to which they are in line with, and can speak to, Butler’s ideas.

Evolutionary psychology is, we would argue, the least compatible with Butler’s view on sex and gender. Evolutionary approaches to the psychology of gender maintain that gender differences are, for the most part, genetic – resulting from the different adaptive problems faced by women and men in their evolutionary past (see Byrd-Craven and Geary, 2013), particularly due to reproductive differences such as paternal uncertainty for men and higher parental investment for women. These differences, it is argued, then shaped our genes – and gender differences – through sexual selection (i.e., gender differences in the factors predicting successful reproduction; Darwin, 1871). These approaches can be described as essentializing gender, that is, promoting the belief that men and women share an important but unobservable “essence.” Essentialism includes a range of factors such the degree to which individuals perceive social categories to be fixed and natural (Roberts et al., 2017) and has been shown to be associated with greater levels of stereotyping and prejudice (Brescoll and LaFrance, 2004; Bastian and Haslam, 2006). Evidence further suggests people who hold highly essentialist beliefs of gender are more supportive of what the authors call “boundary-enhancing initiatives” such as gender-segregated classrooms and legislation forcing transgender individuals to use the bathroom associated with the sex they were assigned at birth (Roberts et al., 2017). Thereby, essentialism, and the resultant stereotypes and prejudice, contribute to the reinforcement of the status quo.

Evolutionary psychology’s approach to gender exemplifies many points Butler (1990) criticizes in *Gender Trouble*. First, it treats sex as a pre-discursive binary fact rather than a cultural construct. In other words, it ignores variability in chromosomes, genitals, and hormones (Fausto-Sterling, 1993; Ainsworth, 2015) and views binary sex – and gender – as an inherent, essential quality. Moreover, evolutionary approaches argue that gender follows from sex and thus portray binary sex as an explanation for, rather than a result of, gender differences (i.e., gender performance). In addition to ignoring the existence of intersex individuals, these approaches also often ignore homosexuality, focusing exclusively on heterosexual desires and reproduction. Thus, we would argue, such evolutionary approaches play into the patriarchal system of compulsory heterosexuality in which women function primarily as mothers and wives.

Social structural approaches to gender such as early conceptions of social role theory (Eagly, 1987) and the stereotype content model (Fiske and Stevens, 1993) are more compatible with Butler’s views. Such approaches argue that societal structures such as social roles and differences in power and status determine gender stereotypes, which affect both gendered behavior as well as reactions to those who deviate from gender stereotypes. In other words, gender stereotypes provide the “script” for the performance of gender with negative consequences for those who fail to “learn their lines” or “stick to the script”.

The social psychological literature provides many empirical examples of these negative consequences. For example, Rudman and colleagues describe how those who deviate from their scripts often encounter backlash in the form of economic and social penalties (for a review see Rudman et al., 2012). This backlash discourages individuals from engaging in stereotype-incongruent behavior as they avoid negative consequences in the future, reducing their potential to act as deviating role models for others. Moreover, witnessing the backlash gender troublemakers encounter may also vicariously discourages *others* from breaking gender stereotypes to avoid negative consequences for themselves. The literature on precarious manhood further suggests that these issues might be particularly pronounced for men (Bosson et al., 2013). Research demonstrates that men must continuously prove their masculinity by avoiding anything deemed feminine to avoid negative consequences such as loss of status. Each of these lines of research are very much in line with Butler’s arguments, both with the idea that those who “fail to do their gender right” are punished and with the idea that the gender binary is a tool to uphold the patriarchy.

However, in other respects, social structural approaches are less compatible with Butler’s arguments. First, they tend not to take non-binary gender into account, and the empirical research tends to operationalize men and women as disjunct categories. Although research focusing on how intra-gender variability is often much larger than between gender variability (e.g., Hyde, 2005) is a good first step, it still ultimately relies on dividing people into the binary categories of female and male. Moreover, these approaches also rarely take issues of intersectionality into account (see Shields, 2008) and focus on stereotypes of white, heterosexual, middle-class, *cis* women and men, although there are some notable exceptions (e.g., Fingerhut and Peplau, 2006; Brambilla et al., 2011).

Approaches from the social identity and self-categorization tradition (Tajfel and Turner, 1979; Turner et al., 1987) view gender as a social identity (e.g., Skevington and Baker, 1989). This tradition argues that in addition to one’s personal identity, different social groups are integrated into the self-concept, forming social identities. These social identities can be based on meaningful social categories such as gender or occupation, but also in response to random allocation to seemingly meaningless groups. The strength of the identification with one’s gender as well the salience of this identity in any given context determine the extent to which the self-concept is affected by gender stereotypes – and in turn the extent to which gendered patterns of behavior are displayed (e.g., Lorenzi-Cioldi, 1991; Ryan and David, 2003; Ryan et al., 2004; Cadinu and Galdi, 2012).

While the idea of gender as an identity – rather than a result of gendered behavior – may be seen as being inconsistent with Butler’s argument, results from minimal group studies (e.g., Tajfel et al., 1971) are very much in line with her reasoning. These studies demonstrate that identities can form on the basis of completely irrelevant, artificial categories and are thus by no means inherent nor inevitable. Thus, while in our given society, these identities are considered to be largely binary, this is not inevitable and likely the result of social forces. Moreover, the evidence from a social identity perspective that supports the notion that changes in context can affect gender salience, levels of identification, and thus the extent of gendered behaviors, are also very much in line with Butler’s arguments.

Lastly, integrative approaches draw on more than one of these traditions as well as developmental, social cognitive, and sociological models of gender. For example, social role theory has developed over time, integrating biological as well as social identity aspects into its framework, resulting in a biosocial approach (Eagly and Wood, 2012). More specifically, more recent versions of the theory argue that the division of labor leads to gendered behavior via three different mechanisms: (1) social regulation (as described above), (2) identity-based regulation, similar to the processes outlined by social identity theory, and (3) biological regulation through hormonal processes such as changes in testosterone and oxytocin. Importantly, these processes interact with one another, that is, hormonal responses are dependent on expectations from others and gender identity. While the social regulation of gender is very much in line with Butler’s arguments, the integration of biological – and particularly evolutionary – perspectives fits less with her idea that gender performance is what creates gender.

Another influential integrative approach is the interactive model of gender-related behavior (Deaux and Major, 1987). Rather than focusing on distal factors which affect gender stereotypes, this model focuses on the situational and contextual factors which result in gendered behavior. The model assumes that the performance of gender primarily takes place in social interactions and serves specific social purposes. Gendered behavior thus emerges based on the expectations held by the perceiver, such as stereotypes, schemata, and knowledge about the specific target; the target themselves (e.g., their self-schema, their desire to confirm or disprove the perceiver’s expectations), and the situation. For example, large gender differences in behavior are likely to emerge when the perceiver believes men and women are very different and thus expects stereotypical behavior, changing the way they treat and communicate with male and female targets; when male and female targets hold very gendered self-schemata and are motivated to confirm the perceiver’s expectations; and when the situation makes stereotypes salient and allows for different behaviors to emerge.

This model is perhaps the most in line with Butler’s perspectives on gender. Similar to Butler, it focuses on the *doing* of gender, that is, on gendered behavior and its emergence in social interactions. Moreover, the model takes a more social cognitive approach, referring to gendered self-schemata rather than gender *identities*. Thus, while retaining the context dependence of gendered behavior inherent in social identity approaches, this model does not necessarily presume gender as a social identity in terms of *men* and *women.* In contrast to all other models discussed above, this model allows for a less binary, more fluid understanding of gender.

While these approaches thus vary considerably in how compatible they are with Butler’s argument, all of them treat gender as a given, pre-existing fact, which is in stark contrast to Butler’s core argument of gender being a performative act, coming into existence only through its own performance. The work of social psychologists operating outside of the experimental framework is more compatible in this regard. More specifically, discourse analysts argue that the self, including the gendered self, is created through language (e.g., Kurz and Donaghue, 2013) and focus on the production of gender in interactions rather than on gender as a predictor of behavior. For example, researchers conducting feminist conversation analysis have examined how patterns in the delivery of naturally occurring speech reproduce heteronormative gender (e.g., Kitzinger, 2005) and research from the ethnomethodology-discursive tradition examines how people acquire a gendered character through speech (e.g., Wetherell and Edley, 1999).

## Future Research Directions

In the previous section, we have outlined how some of the issues raised by Butler, such as the negative reactions to those who fail to do their gender right, have already received considerable attention in the social psychological literature. Other aspects of her argument, however, have received very little attention and hold the potential for interesting future research. We identify two broad ways in which Butler’s work can inform and shape future social psychological research: (a) engendering new research questions which have not yet been investigated empirically, and (b) challenging our way of studying gender itself.

### New Research Questions

Butler’s work is purely theoretical and thus many of her ideas have not been tested empirically, particularly using an experimental approach. Perhaps the most central question that can be examined by social psychologists is whether creating “gender trouble” by subverting ideas about sex, gender, and sexual desire, can indeed lead to changes in binary views of sex and gender and the proscriptive and prescriptive stereotypes that come with these views. Based on predictions derived from social role theory (Eagly, 1987), we would indeed expect that a decrease in the performance of gender as binary (i.e., less gendered social roles) would lead to decreases in gender stereotyping and the reliance on gender as a social category. In other words, if genders are not tied to specific social roles (or vice versa), they lose their ability to be informative, both in terms of self-relevant information (“what should I be like?”) and in terms of expectations of others (“what is this person like?”).

On the other hand, as gender identity is very central to the self-image of many people (Ryan and David, 2003), challenging ideas about gender may be perceived as threatening. Social identity theory and self-categorization theory (Tajfel and Turner, 1979; Turner et al., 1987) argue that members of groups – including men and women – have a need to see their own group as distinct from the outgroup. If this distinctiveness is threatened, highly identified men and women are likely to enhance the contrast between their ingroup and the outgroup, for example by presenting themselves in a more gender stereotypical way and applying stereotypes to the other group (Branscombe et al., 1999) or by constructing gender differences as essential and biological (Falomir-Pichastor and Hegarty, 2014). These identity processes may thus reinforce a system of two distinct genders with opposing traits, and further punish and alienate those who fail to conform to gender norms and stereotypes. Future research needs to investigate the circumstances under which gender trouble can indeed lead to less binary views of gender, and the circumstances under which it does not. This needs to include identifying the psychological mechanisms and barriers involved in such change.

Importantly, this investigation should go beyond examining reactions to women and men who behave in counter-stereotypical ways, such as women in leadership positions or stay-at-home fathers, and include a focus on more radical challenges to the gender binary such as non-binary and *trans* individuals or drag performers. Butler discusses drag as an example of gender trouble in detail, quoting the anthropologist Newton (1968) in her observations of how drag subverts notions of gender. Discussing “layers” of appearance, Newton remarks that on the one hand, the outside appearance of drag queens is feminine, but the inside (i.e., the body) is male. At the same time, however, it appears that the outside appearance (i.e., body) is male, but the inside (the “essence”) is feminine, making it hard to uphold consistent, essentialist ideas about sex and gender. Butler further argues that the exaggeration of femininity (in the case of drag queens) and masculinity (in the case of drag kings) in drag performances highlights the performative nature of gendered behaviors, that is, how gender is created through gendered performance. On the other hand, we would argue that because drag performances often draw heavily on gender stereotypes, they may also reinforce the idea of what it means to be a man or a woman. To our knowledge, there is no psychological research on how drag affects perceptions of gender, but as drag becomes more and more accessible to a wider, and more mainstream, audience (e.g., due to popular TV shows such as RuPaul’s Drag Race) it might be an enlightening line of research to pursue. Does drag indeed highlight the performative nature of gender or does it simply reinforce stereotypes? Are reactions to appearance-based disruptions of the gender binary different to behavior-based ones such as reactions to assertive women or submissive men?

Another potential line of research to pursue would be to build on the discursive literature by examining the performative nature of gender from an experimental social psychological perspective, testing how gender is created through speech and behavior. Drawing on some of the findings from qualitative psychological research discussed in the previous section might be helpful in developing predictions and quantitatively testable hypotheses.

Finally, if gender trouble is indeed effective in challenging binary, essentialist views of sex and gender, it is worth investigating how disruptive gender performance can be encouraged and used as a means of collective action. The literature on collective action to achieve gender equality has often drawn on (gender) identity-based ideas of mobilization (e.g., Kelly and Breinlinger, 1995; Burn et al., 2000). As outlined above, Butler criticizes these approaches and argues that group-based identities (“we, women”) are not necessary to achieve change. How then can we inclusively mobilize others to engage in collective action without drawing on gender identities and inadvertently reinforcing the gender binary – and with it the patriarchal system of compulsory heterosexuality it supports?

More recently, psychologists have argued that it might be more effective to focus on “feminist” (rather than gender) ideologies which acknowledge, rather than ignore, issues of intersectionality (see Radke et al., 2016), and to encourage men to engage in collective action to achieve gender equality (e.g., Subašić et al., 2018). We agree with these arguments but further suggest that collective action research should examine how individuals of *any* gender can (a) be motivated to engage in collective action to achieve gender equality generally, and (b) be motivated to engage in *gender trouble* and disrupt binary notions of gender as a form of collective action.

### Studying Gender From a Performative Perspective

In addition to new research question, Butler’s work also highlights the need for different methodological approaches to gender in experimental social psychology, and indeed there is much that could be learnt from those that work in the discursive tradition. There is also the potential for gender researchers to engage in gender trouble themselves by changing the way in which they treat gender.

For the most part, experimental psychologists have tended to examine gender as a predictor or independent variable – examining gender differences in all manner of social, cognitive, and clinical measures (e.g., Maccoby and Jacklin, 1974; Hyde, 2005). Indeed, as researchers, we (the authors) are guilty of publishing many papers using this methodology (e.g., Haslam and Ryan, 2008; Morgenroth et al., 2017). Similar to performative speech acts, we would argue that this can be seen as a performative research practice. The way in which we conduct our research and the choices we make in relation to gender creating the very construct that is studied, namely gender and gender differences. Our assumptions of gender as binary, pre-discursive, and natural produces research that focuses on binary, categorical gender as a predictor of gendered attitudes and behavior.

However, to our knowledge, there is very little quantitative or experimental research, that looks at the psychological processes implicated in the *performance* of gender, that is, treating gender as an outcome or dependent variable. If experimental social psychologists are to contribute to gender trouble, we should shift our views away from sex and gender as *causes* for behavior and psychological outcomes (i.e., as an independent or predictor variables). Instead, we should treat gender – whether measured as an identity, in terms of self-stereotyping, as simple self-categorization – as a *result* of societal and psychological forces. Rather than asking what sex and gender can explain, we need to look at what explains sex and gender.

Moreover, while the literature acknowledges that gender salience and gender self-stereotyping vary depending on context (e.g., Lorenzi-Cioldi, 1991; Ryan and David, 2003), gender itself, regardless of how it is measured, is measured as a stable, and discrete construct. One *is* a man or a woman and remains so over the course of one’s life. If, however, we view gender as a performance, then we must also view gender as an act, a behavior, which changes depending on context and audience. Asking participants to tick a box to indicate one’s gender – as many of us often do in our research practices – is an overly simplistic measure and cannot capture the nuances of *doing gender*. It is neither informative nor, we would argue, terribly interesting. Instead, one could measure gender identity salience and importance or gender performance – for example measuring gender stereotypical behavior or other types of gendered self-stereotyping (e.g., using measures similar to the Bem Sex-Role Inventory; Bem, 1974).

Similarly, we, as researchers, need to stop treating gender as a binary variable. This includes our research practices as well as our theory development and research communications. For example, the demographic sections of most questionnaires should not restrict gender to two options. Instead, they should either provide a range of different options (e.g., non-binary, genderqueer, genderfluid, and agender) or allow open responses. We would also suggest not using the option “other” in addition to “male” and “female” as it can be perceived as stigmatizing. Similarly, if asking about sex rather than gender, at least a third option (i.e., intersex) should be provided (see Fonesca, 2017, for examples).

However, we need to go beyond that. At the moment, even when gender is measured in a non-binary way, those who fall outside of the gender binary are usually excluded from analysis. This is equally true for sexual minorities. Unless sexual orientation is central to the research question, those who don’t identify as heterosexual are often excluded by gender researchers as stereotypes and norms of gay, lesbian, bisexual, or asexual individuals often differ from general gender stereotypes. While these decisions often make sense for each individual case (and we, the authors, have in fact engaged in them as well), this overall produces a picture that erases variation and reinforces the idea that there are two opposing genders with clear boundaries. As experimental social psychologists with an interest in gender, we need to do better. Similarly, our theories themselves should allow for a fluid understanding of gender which also takes issues of intersectionality – with sexual orientation, but also with race, class, and other social categories – into account.

Finally, when we talk about gender, we should do so in a way that makes gender diversity visible rather than way that marginalizes non-binary gender further. For example, replacing binary phrases such as “he or she” with gender-neutral ones such as “they” or ones that highlight non-binary gender such as “he, she, or they” or “he, she, or ze”^3^. While the use of the gender-neutral singular “they” is often frowned upon and deemed grammatically incorrect (American Psychological Association, 2010; University of Chicago, 2010), it has in fact been part of the English language for centuries and was widespread before being proscribed by grammarians advocating for the use of the generic masculine in the 19th century (Bodine, 1975). Despite these efforts, the singular “they” has remained part of spoken language, where it is used to refer to individuals whose sex is unknown or unspecified (“Somebody left *their* unicorn in my stable”) and to members of mixed-gender groups (e.g., “Anybody would feed *their* unicorn glitter if *they* could”).

The use of new pronouns such as “ze,” specifically developed to refer to people outside of the binary, might be more effortful and equally controversial. However, evidence from Sweden, where the gender-neutral pronoun “hen” has become more widely used since the publication a children’s book using only “hen” instead of “han” (he) and “hon” (her) in 2012, indicates that attitudes toward its use have shifted dramatically from predominantly negative to predominantly positive in a very short amount of time (Gustafsson Sendén et al., 2015). As gender researchers, we should be at the forefront of such issues and promote and advance gender equality – and gender diversity – not only through our research but also by communicating our research in a gender-inclusive way, especially in light of Butler’s (and others’) arguments that language is a crucial mechanism in creating gender and reinforcing the gender binary.

## Conclusion

In this paper we put forward suggestions for ways in which Judith’s Butler’s (1990) notions of gender trouble could be integrated into experimental social psychology’s understanding of gender, gender difference, and gender inequality. We have outlined her work and discussed the extent to which prominent views of gender within psychology are compatible with this work. Moreover, we suggested potential avenues of future research and changes in the way that we, as researchers, treat gender.

We believe that, as experimental social psychologists, we should be aware that we may inadvertently and performatively reinforce the gender binary in the way in which we do research – in the theories we develop, in the measures that we use, and in the research practices we undertake. By taking on board Butler’s ideas into social psychology, we can broaden our research agenda – raising and answering questions of how social change can be achieved. We can provide a greater understanding of the psychological processes involved in creating gender trouble, and in resisting gender trouble – but above all, we are in a position to create our own gender trouble.

## Notes

The first author of this paper uses they/them/their pronouns, the second author uses she/her/hers pronouns.

## Author Contributions

TM and MR jointly developed the ideas in the paper. TM wrote the paper. MR read the paper and provided feedback on several drafts of the paper.

## Conflict of Interest Statement

The authors declare that the research was conducted in the absence of any commercial or financial relationships that could be construed as a potential conflict of interest.

## Footnotes

1 Please note that these terms are based on the common view of naturally binary sex under which most researchers operate. We do not mean to imply that Butler herself would use these terms or, indeed, would be convinced by the idea that these bodies – or any bodies – exist “naturally” prior to interpretation.

2 “*Cis*” refers to individuals for whom the sex they are assigned at birth and their gender identity align.

3 The exact origins of the non-binary pronouns ze/hir or ze/zir are unknown, but ze/hir is often credited to Bornstein (1996). There are no clear conventions around non-binary pronoun use and many different alternatives have been proposed.
